# Biosynthetic labeling with 3-O-propargylcaffeyl alcohol reveals in vivo cell-specific patterned lignification in loquat fruits during development and postharvest storage

**DOI:** 10.1038/s41438-021-00497-z

**Published:** 2021-03-10

**Authors:** Nan Zhu, Chenning Zhao, Yuqing Wei, Chongde Sun, Di Wu, Kunsong Chen

**Affiliations:** College of Agriculture and Biotechnology/Zhejiang Provincial Key Laboratory of Horticultural Plant Integrative Biology/The State Agriculture Ministry Laboratory of Horticultural Plant Growth, Development and Quality Improvement, Zhejiang University, Zijingang Campus, 310058 Hangzhou, P. R. China

**Keywords:** Secondary metabolism, Non-model organisms

## Abstract

Lignification is a major cell wall modification that often results in the formation of sophisticated subcellular patterns during plant development or in response to environmental stresses. Precise localization of the spatiotemporal deposition of lignin is of great importance for revealing the lignification regulatory mechanism of individual cells. In loquat fruits, lignification typically increases the flesh lignin content and firmness, reducing their edibility and processing quality. However, the precise localization of the spatiotemporal active zones of lignification inside loquat fruit flesh remains poorly understood, and little is known about the contribution of patterned lignification to cell wall structure dynamics and the subsequent fruit-quality deterioration. Here, we performed an emerging bioorthogonal chemistry imaging technique to trace the in vivo patterned lignification dynamics in cells of loquat fruit flesh during development and storage. In developing fruits, lignified cells (LCs) and vascular bundles (VBs) were the zones of active lignification, and ring-like LCs deposited lignin at both the inner wall layer and the outer periphery sides. The domino effect of the generation of LCs was preliminarily visualized. In mature fruits, the newly formed lignin in the flesh of fruits during storage was specifically deposited in the corners and middle lamellae of parenchyma cells surrounding the VBs, resulting in the development of a reticular structure. Based on the findings, distinct spatiotemporal patterned lignification modes for different flesh cells in loquat fruits were proposed. These findings provide loquat lignification dynamics together with spatiotemporal data that can improve our understanding of the lignification process *in planta*.

## Introduction

Cell walls are highly complex configurations of structurally diverse polysaccharides and execute essential functions, including constraining cell expansion, maintaining turgor pressure, and providing plants with mechanical strength and protection^1^. In addition to their fundamental functions, some cell walls with unique composition and architecture in different plant tissues and organs develop to perform specific functions during plant growth and development^2^, such as ensuring the precise separation of floral organs^3^, releasing functional pollen^4^, and promoting the efficient formation of lateral roots^5^. Lignin, which comprises a type of cross-linked phenolic polymers accounting for 15–30% of lignocellulose, is a major structural component of cell walls in plants. Generally, lignin fills the cell wall spaces among cellulose, hemicellulose, and pectin components; covalently cross-links different plant polysaccharides, and thereby imparts cell walls with mechanical strength, rigidity, imperviousness, and resistance to pathogens^6^. In addition, patterned lignin deposition is a major cell wall modification in which certain cell types and tissues are formed, and this process plays essential roles in plant development or in response to various environmental stresses^3,7^.

The textural properties of fruits and vegetables, which affect taste and influence consumer appeal, are closely related to the metabolism and structural changes of cell wall components^8^. Softening and lignification are two main kinds of texture variation of fruits during ripening and postharvest storage. Softening commonly occurs in many fruits, such as tomato^9^, peach^10^, and apple fruits^11^. The progressive solubilization and depolymerization of pectic polysaccharides, which results in cell wall disassembly and loss of cell structure, is considered to be responsible for the softening of many fruits^12–14^. For example, using engineered genetically modified tomato plants, Uluisik et al. showed that rapid fruit softening was due to the breakdown of cross-linked HG polymers in the middle lamellae and tricellular junctions, which enables further degradation of pectic polysaccharides in the cell wall^9^. In contrast to softening, lignification occurs in fruits of several kinds of species, such as loquat^15,16^, kiwifruit^17^, and mangosteen^18^, and is considered an unusual phenomenon. The most notable case is the substantial flesh lignification of loquat fruit during postharvest storage.

Loquat (*Eriobotrya japonica* Lindl.) fruits are rich in bioactive compounds and are popular among consumers. However, loquat fruits suffer rapid senescence and deterioration after harvest^19^. Low-temperature storage is an effective technique for extending the postharvest life of loquat fruits but results in substantial flesh lignification^20^. The symptoms of flesh lignification include lignin accumulation, increased fruit firmness, stuck peels, leathery pulp, and juiceless pulp, which inevitably affect fruit edibility and processing quality^21^. Lignin accumulation and flesh hardening are the most typical physiological disorders of loquat fruit lignification^15^. Our previous work revealed that the accumulation of lignin is significantly correlated with an increase in fruit flesh firmness (*r* = 0.95**)^19^. In terms of molecular regulation, we also found that the expression of lignin biosynthesis-related genes (*PAL*, *4CL*, and *CAD*) increased and identified their transcription factors (*NAC* and *MYB*) in loquat fruit during postharvest lignification^16,22^. These physicochemical and molecular biological studies have promoted the understanding of the mechanism underlying loquat lignification at the homogenized bulk tissue and molecular levels. However, there is still a gap in knowledge between flesh physicochemical disorders and molecular characterization. The contribution of lignin accumulation to cell wall structure variation and the subsequent increase in flesh firmness remain poorly understood. However, little is known about the single-cell-level mechanism of lignification dynamics in loquat fruits during development and postharvest storage.

Precise localization of the spatiotemporal deposition of lignin can provide critical insight into the morphologic mechanism of cell walls of normal or specialized structures and thereby allows more insight into the relationship between cell wall structure and cell function. Various microscopy strategies have been employed to plot the distribution of lignin and examine its compositional and molecular structural properties in plant cell walls in situ^23,24^. Vibrational spectroscopy-based imaging techniques, mainly Raman and infrared microspectroscopy, are considered very powerful and are highly recommended for in situ lignin and cell wall characterization^25^. Vibrational microscopy has been widely applied to nondestructive probe in situ lignin chemistry in the cell walls of native-state^26^ as well as genetically modified^27^ and preprocessed plants^28^. In addition, several other imaging strategies, such as classic light microscopy histochemistry^29^, (auto)fluorescence imaging^30^, mass spectrometry imaging^31^, and transmission electron microscopy^32^, have shown their respective strengths for the visualization of lignin in plant cell walls. However, these imaging techniques are not sufficient for exclusively distinguishing the newly deposited lignin in vivo during a particular time period or that induced in response to specific stress from preexisting lignin; most of the time, these techniques are used to detect the distribution of total lignin in cells. Given that lignin deposition is very heterogeneous among different kinds of cells and tissues in plants^33^, it is necessary to trace the precise spatiotemporal deposition of new lignin during a particular time period or that induced in response to specific stress to define the active lignified cells and tissues. Further molecular research targeting the specific cells and tissues of active lignification rather than using bulk plant tissues can then be performed. This allows unbiased insights into the relationships between the lignification process and the environmental influences.

The bioorthogonal chemical reporter approach has recently demonstrated success in the direct tracing of the spatiotemporal process of lignification in living plants^34–36^. This methodology uses chemical reporter-tagged monolignol mimics as precursors for metabolic incorporation into lignin and subsequently introduces a detectable tag for visualization^37,38^. By the use of this methodology, precise spatial information on the zones of active lignification and heterogeneity in single-cell lignification dynamics has been revealed. However, previous studies have typically focused mainly on demonstrating a strategy of developing clickable monolignol mimics and verifying their biocompatibility with lignification in plant species such as Arabidopsis and flax. In this study, in the face of the agricultural industry problems involving fruit lignification, we applied this emerging imaging strategy using bioorthogonal chemical labeling aiming to provide a spatiotemporal aspect to studying the deposition of lignification and exploring the cellular mechanism underlying loquat fruit lignification. Here, the spatiotemporal patterned deposition of lignin in loquat fruit flesh cells during development and postharvest storage was directly traced using biosynthetic labeling with 3-O-propargylcaffeyl alcohol (CA-Alk). First, we demonstrated CA-Alk biocompatibility with lignification using Arabidopsis and Nicotiana and revealed the patterned deposition of lignin in their stem sections. The precise microscopic zones of active lignification in the flesh cells of loquat fruits during different stages were then plotted. Lignification was found to vary among different flesh cells, and specific patterns of cell wall structures had formed. Finally, we proposed cellular modes for the lignification process in different flesh cells in loquat fruits during development and postharvest storage and discussed the relationship between the increased lignin content and the increase in fruit firmness and rough taste.

## Results

### Compatibility of 3-O-propargylcaffeyl alcohol with lignification *in planta*

Coniferyl alcohol (CA) is one of the main building blocks of lignin and is regarded as a model precursor in lignin biosynthesis in plants. In this study, we synthesized 3-O-propargylcaffeyl alcohol (CA-Alk), a coniferyl alcohol analog, according to the methods of Bukowski et al.^37^ to probe the lignification process *in planta*. Supplementary Fig. S1 shows the chemical structures of both natural CA (Supplementary Fig. S1a) and the synthesized CA-Alk, which contains a bioorthogonal alkynyl functional group at the 3-O-position (Supplementary Fig. S1b). We then collected and compared the fingerprint Raman spectra of CA and CA-Alk. CA-Alk showed an additional significant scattering peak centered at 2128 cm^−1^ (Supplementary Fig. S1c), which originated from the alkynyl stretching vibration. In addition, the molecular structure of CA-Alk was verified based on its nuclear magnetic resonance spectrum (Supplementary Fig. S1d).

**Fig. 1:**
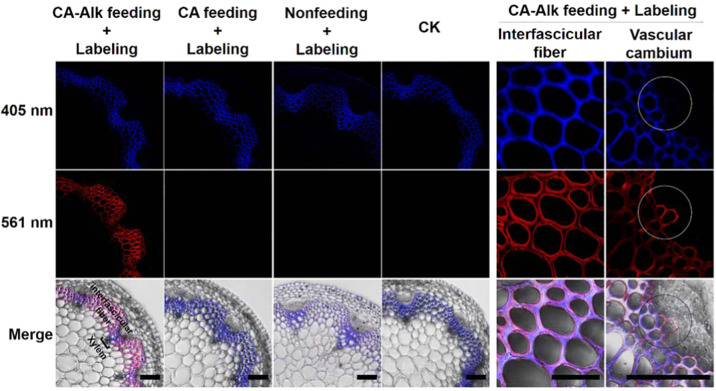
Incorporation of CA-Alk into stem sections of 6-week-old Arabidopsis. The newly deposited lignin (561 nm, click labeling) was successfully highlighted and could be distinguished from the preexisting lignin (405 nm, lignin autofluorescence) in the stem sections after feeding with CA-Alk. The CA feeding and labeling, nonfeeding, and labeling and CK (native state) sections showed no 561-nm signal. The 561-nm signals mainly localized in the inner wall layers of the interfascicular fiber cells and some vascular cambium cells. Three biological replicates were evaluated. At least three replicate sections were imaged per biological replicate under each treatment condition. Scale bar, 50 μm

Next, to demonstrate the reliability of the synthesized CA-Alk biocompatibility with lignification *in planta*, feeding experiments with CA-Alk in Arabidopsis and Nicotiana stem sections were performed. After feeding with CA-Alk and labeling with Alexa 594-azide, the stem sections showed strong fluorescence at 561 nm compared with those of the other groups, including the CA feeding and labeling, nonfeeding and labeling, and nonfeeding and nonlabeling groups; moreover, all Arabidopsis stem sections showed lignin autofluorescence (405-nm excitation) in the vascular bundles (Fig. 1). This indicates the successful incorporation of CA-Alk into the newly formed lignin, and therefore, the newly deposited lignin (561 nm) in Arabidopsis stems during incubation could be highlighted and distinguished from the preexisting lignin (405 nm). Moreover, the fluorescence at 561 nm was found mainly in the interfascicular fiber cells, indicating that these cells were the zones of active lignification in Arabidopsis stems during incubation. With respect to microscopy observations, the newly formed lignin was deposited in the inner wall layers of the interfascicular fiber cells and some vascular cambium cells. The above results suggest that the synthesized CA-Alk was successfully incorporated into lignified cell walls and that the spatial details of lignification in Arabidopsis stems could be traced.

For Nicotiana, another model plant species, except for the successful imaging of new lignin (Supplementary Fig. S2), some differences in the spatial details of lignification among different cells in the stem sections were found (Supplementary Fig. S3). In xylem cells, new lignin was mainly deposited in the inner wall layer, whereas in the tracheids, not only the inner wall layer but also the outer periphery were active in lignification during incubation. Taken together, these results show that the deposition pattern of lignin varied among different cell types and suggest that different lignification mechanisms occur among them. Like in Arabidopsis stem sections, lignin deposition in vascular cambium cells was also visualized in Nicotiana stem sections. The cambium is a meristematic layer located at the periphery of stems and is responsible for extended stem thickening^39^. Here, we demonstrated that the biosynthetic labeling strategy was an alternative competent technique for visualization of the lignification of cambium cells.

### Direct tracing of lignification dynamics in cells of flesh cubes in developing loquat fruits

Flesh lignification in loquat hardens fruits and reduces their quality, but the mechanism underlying flesh lignification at the single-cell level remains poorly understood. Our previous study showed that a kind of high-lignin-content lignified cell is present in loquat fruits at harvest^40^. This indicated that lignin deposition began during the developmental stages of the loquat fruits. To obtain a comprehensive and visual understanding of the lignification dynamics throughout the life of loquat fruits, including their on-tree growth and postharvest storage, we performed direct tracing of lignin deposition among different cells in cubes of loquat flesh of developing and postharvest fruits.

**Fig. 2:**
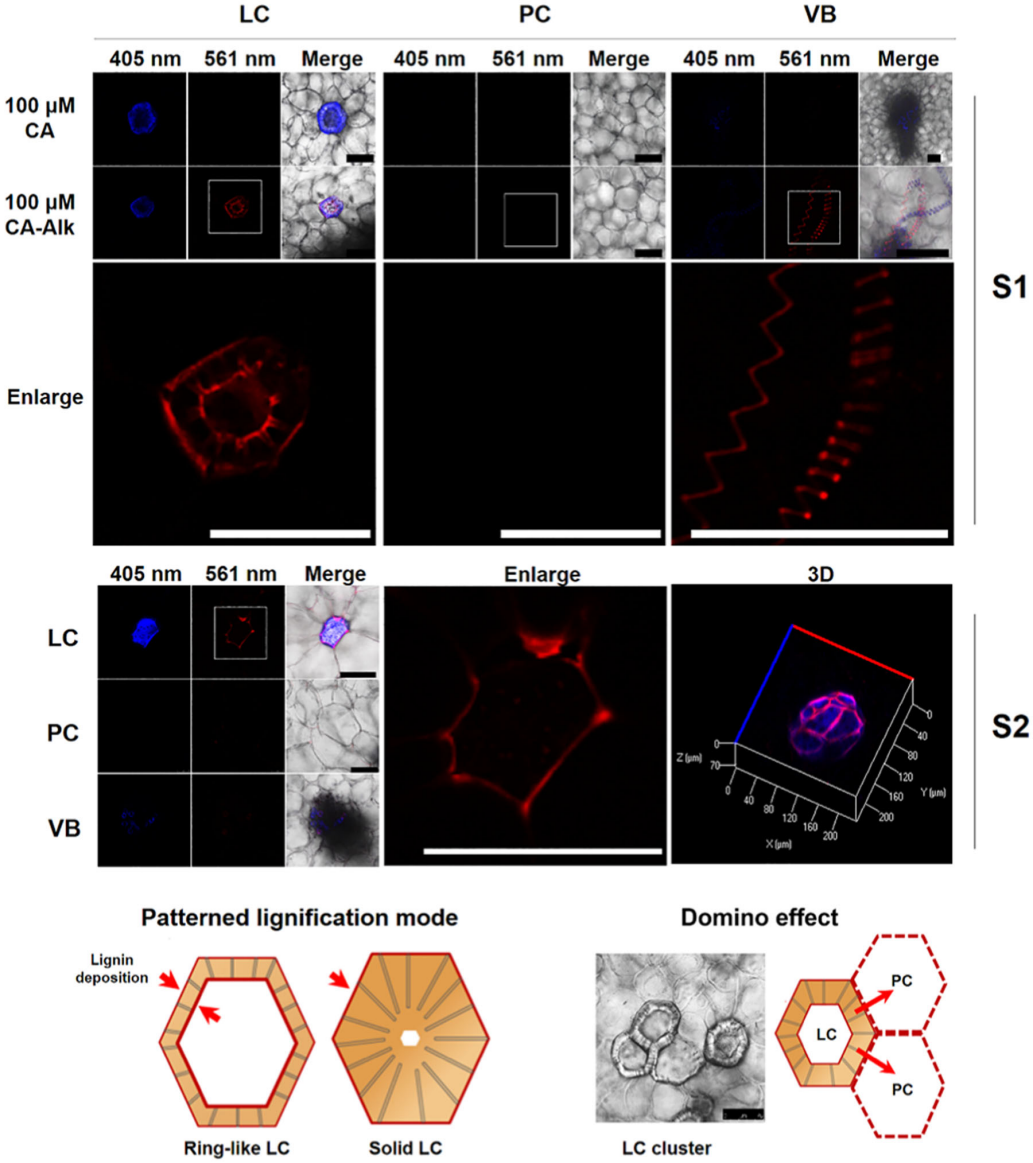
Patterned deposition of lignin among different flesh cells in S1- and S2-stage loquat fruits. Cubes of the flesh of S1- and S2-stage loquat fruits were incubated together with 100 μM CA-Alk at 25 °C for 24 h. After sectioning and click labeling, the LCs and VBs in the cubes of the flesh of S1-stage fruits showed a 561-nm fluorescence signal. In the cubes of the flesh of S2-stage fruits, some LCs became solid, but their outer periphery regions showed a 561-nm fluorescence signal (the enlarged view and 3D picture). A pattern of LC lignification mode and a domino effect on the development of LCs were proposed. Three biological replicates were included. At least three replicate sections were imaged per biological replicate under each treatment condition. Scale bar, 75 μm

When fruitlets (S1) were taken as examples, three kinds of cells were observed in the loquat flesh, i.e., vascular bundle (VB) cells, parenchyma cells (PCs), and lignified cells (LCs) (Supplementary Fig. S4). In the flesh of the loquat fruitlets (S1), LCs and spiral vessels in the VBs are active in lignin deposition during incubation (Fig. 2). An enlarged view of LCs in loquat fruitlet flesh shows that both the inner wall layer and the outer periphery of LCs deposit newly formed lignin. These results indicate that lignin is mainly synthesized to form LCs and VB vessels at the growing stage of fruitlets (S1). At the turning stage (S2), though some of the LCs become solid, they are still the main active zones of lignification. As shown in the enlarged view of LCs in turning-stage fruits (S2), the outer periphery regions of LCs can continue depositing lignin during incubation. However, unlike the tracheid cells in Nicotiana stems, where the active zones of lignification are tightly adjacent to the primary wall, the active lignification zones in the LCs seem to be positioned in the walls of their adjacent parenchyma cells. From the 3D picture showing the patterned deposition of lignin in the LCs, we can see that the contact regions of the LCs and their neighboring parenchyma cells are highlighted as lignified active zones. Considering that some of the lignified cells exist in pairs or clusters, we believe that the generation of lignified cells has a domino effect in loquat fruits during development. Lignified cells have the ability to cause their neighboring parenchyma cells to lignify in loquat fruits during development. The above results, on the one hand, show the lignification dynamics in the flesh cells of the developing fruits (S1 and S2). On the other hand, flesh lignification has already occurred in the developmental stages before harvest.

### Direct tracing of the patterned deposition of lignin in loquat cells during storage

**Fig. 3:**
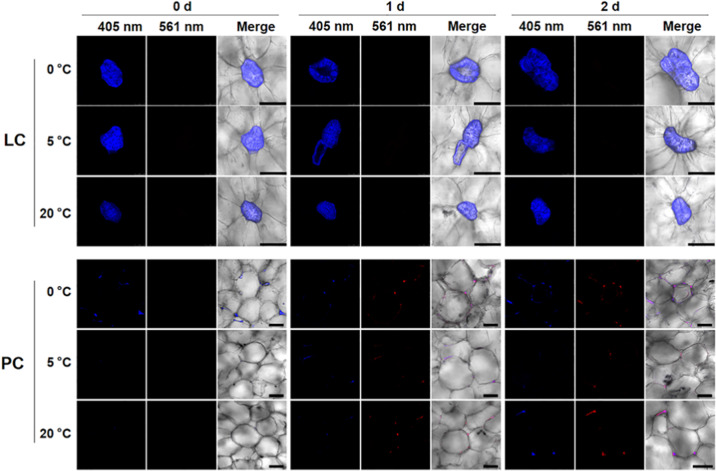
Tracing newly deposited lignin in cubes of the flesh of S3-stage loquat fruits during storage. Cubes of the flesh of S3-stage fruits were incubated with 100 μM CA-Alk at 0, 5, and 20 °C for 2 days. After sectioning and click labeling was performed, no 561-nm fluorescence signals were detected in or around the LCs, but 561-nm fluorescence signals were detected in some corners and middle lamellae of PCs. Three biological replicates were included. At least three replicate sections were imaged per biological replicate under each treatment condition. Scale bar, 150 μm

The flesh lignification process during postharvest storage results in an increase in fruit firmness and rough texture. Even a positive correlation between increased lignin content and increased fruit firmness was found in Luoyangqing loquat fruits during postharvest storage^15^. Here, using feeding experiments, we plotted the patterned deposition of lignin in cubes of the flesh of mature loquat fruits stored at different temperatures (0, 5, and 20 °C) for 2 days. These three temperatures were used because 0 and 20 °C correspond to the temperature conditions for chilling injury-induced and natural senescence lignification in loquat fruits during postharvest storage, respectively, whereas 5 °C can alleviate lignification to some extent^41^. As shown in Fig. 3, no newly deposited lignin was found in or around the LCs during the feeding period for 2 days at 0, 5, or 20 °C. This result indicates that, unlike those in developing loquat fruits, the LCs in the mature loquat fruits did not deposit lignin during postharvest storage for 2 days. However, in some parenchyma cells, newly deposited lignin was detected at temperatures of 0, 5, and 20 °C. The newly formed lignin was trapped in the cell corners and the middle lamellae of the parenchyma cells.

**Fig. 4:**
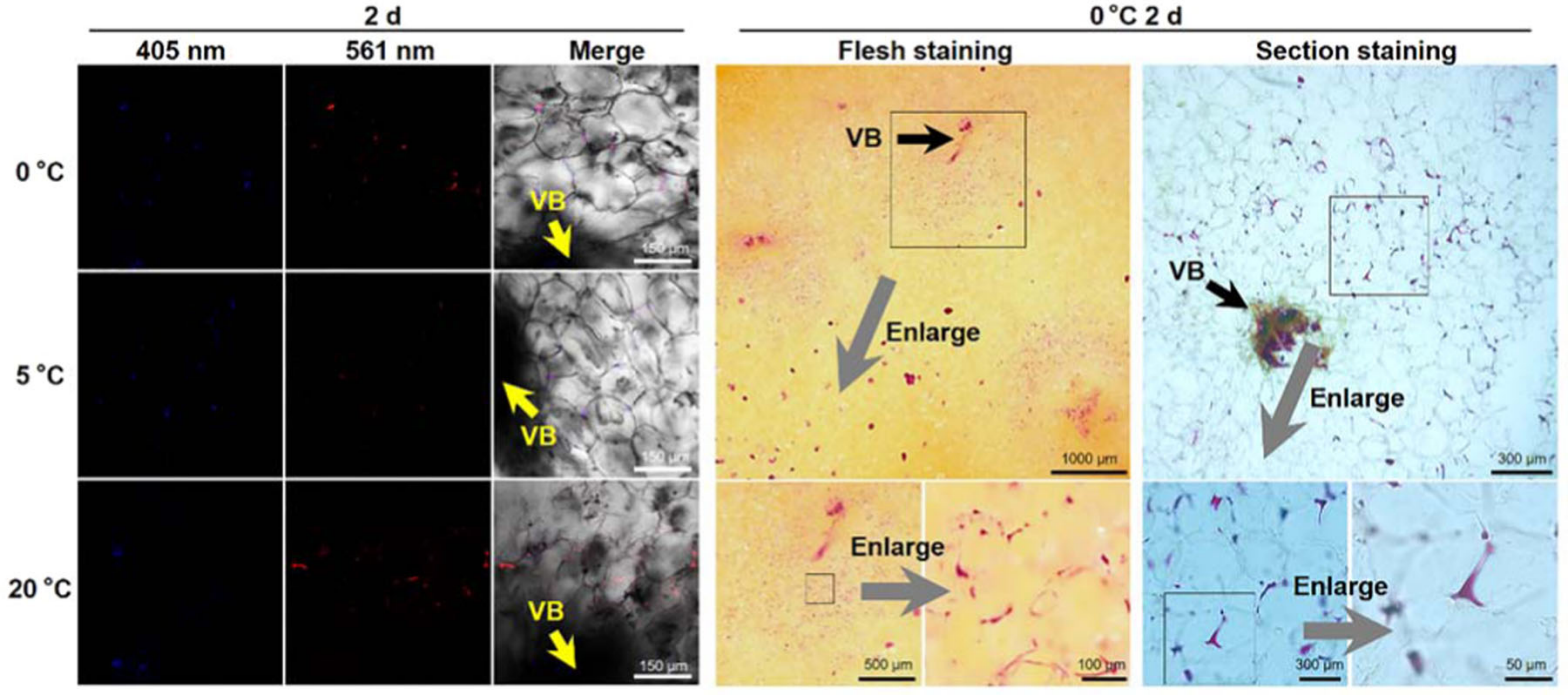
Specific click labeling and lignin staining showing the patterned deposition of lignin in cubes of the flesh of S3-stage loquat fruits during storage. The newly formed lignin (561 nm) was specifically deposited in the corners and middle lamellae of the PCs surrounding the VBs. Both the flesh lignin staining and flesh microscopy section staining images show that the VB-surrounding PCs were lignified after 2 days of storage at 0 °C. The corners and middle lamellae of the PCs deposited lignin. Three biological replicates were included. At least three replicate sections were imaged per biological replicate under each treatment condition

Though it was determined that the cell corner and the middle lamella regions of the parenchyma cells deposited newly formed lignin during incubation, not all the parenchyma cells in the fruit flesh performed such deposition. As shown in Fig. 4, in the large-scale view, it can be seen that the parenchyma cells around the vascular bundles show the 561-nm signal, indicating deposition of new lignin in these parenchyma cells during incubation. Therefore, we suppose that the cell corners and middle lamellae of the parenchyma cells around the vascular bundles are the zones of active lignification in loquat fruits during postharvest storage. This speculation was confirmed according to phloroglucinol–HCl lignin staining at multiple scales in cold-stored loquat fruits. Direct lignin staining of the loquat fruit flesh showed that the parenchyma cells surrounding vascular bundles were lignin- stained, whereas the other flesh was not stained, except for some lignified cells. Microscopy examination of the lignin staining in the flesh section also showed that the parenchyma cells around the vascular bundle have lignin deposition, and the deposited lignin is localized in the cell corner and the middle lamella regions.

## Discussion

Texture change is an inevitable problem faced by the fruit industry. The texture is one of the major determinants of fruit quality, including fruit transportability, storability, and shelf life, and influences consumer preference. Cell wall dynamics play key roles in the processes during fruit texture change^13^. Recent studies have provided insights into the physiological and molecular mechanisms underlying cell wall modifications involved in fruit textural change^42^. In particular, the softening of many fruits is mainly attributed to the disassembly of cell wall polymers and the dissolution of the middle lamella promoted by the coordinated action of cell wall-degrading enzymes or proteins^12,43^. In contrast, lignification in loquat fruits leads to the opposite trend, which results in hardening of the fruits and causes undesired physiological disorders such as peel sticking and leathery and juiceless pulp. In the process of loquat lignification, the expression of lignin biosynthesis-related genes is active, and the lignin content increases, which is considered the main reason for the increase in flesh firmness^15,44^. However, there are very few studies that have examined the precise spatiotemporal localization of newly formed lignin, and the effects of lignin deposition on the formation and structure of cell walls in the flesh of postharvest loquat fruits and the cellular mechanism underlying lignification dynamics in loquat fruits remain elusive. Such an investigation would provide a spatiotemporal aspect to lignification and help promote our understanding of the relationship between lignin deposition and the increase in fruit flesh firmness in terms of cell wall structure and function.

In the face of the agricultural industry problem of fruit lignification, we applied an emerging imaging strategy based on metabolic click labeling to study the cellular mechanism underlying lignification in loquat fruits. We first used a modified lignin monomer to investigate lignification in the stems of Arabidopsis and Nicotiana model plant species, and patterned deposition of lignin among different cell types was successfully revealed. We then performed direct tracing of the spatiotemporal process of the specific deposition of lignin in loquat flesh during fruit development and postharvest storage. We plotted the patterned deposition of lignin in loquat fruit flesh cells and demonstrated that the lignified cells were the zones of active lignification in fruits during development, whereas during postharvest storage, the newly formed lignin was specifically deposited in the corners and middle lamellae of some parenchyma cells around the vascular bundles.

Here, on the basis of our findings, we proposed a domino effect for the generation of lignified cells in developing loquat fruits. Lignified cells pass the lignification signal to their neighboring parenchyma cells, which could explain why lignified cells usually exist in clusters. Typically, in pear fruits, stone cells always exist in clusters, which contribute to poor fruit quality^45^. However, visual evidence, as well as investigations of the mechanism underlying the formation of these cell clusters, currently remains scarce. With respect to the lignification of Arabidopsis stems, a “good neighbor” hypothesis has been proposed in which neighboring nonlignified cells contribute to lignification zones^46^. Recently, Smith et al. revealed that during development, neighboring xylem fibers and parenchyma cells produce monolignols and subsequently contribute to TE lignification^47^. The “good neighbor” hypothesis of Arabidopsis stem lignification could support the domino effect of lignified cells in loquat fruits to a certain extent. However, some differences still exist. In Arabidopsis, xylem parenchyma cells do not develop a lignified cell wall per se, in which case lignification undergoes noncell autonomous lignification^46^. In loquat, neighboring parenchyma cells might first contribute to the noncell autonomous lignification of lignified cells, and as lignification progresses, neighboring parenchyma cells harden and become lignified cells per se, ultimately forming lignified cell clusters. These lignified cell clusters initially originate from one or very few lignified cells and gradually expand. Therefore, we called this a domino effect. It should be noted, though, that the domino effect of the lignified cells was found only in developing loquat fruits.

When loquat fruits are mature, flesh lignification process differs from that in developing loquat fruits. In this study, we used mature cubes of flesh subjected to different temperatures for 2 days to simulate the lignification of loquat fruits during postharvest storage. Previous studies have shown that lignification is obvious in the first 2 days during storage^48^. During incubation for 2 days, the zones of active lignification were localized in the corners and middle lamellae of parenchyma cells rather than lignified cells, which are active in lignification in the flesh of developing loquat fruits. Interestingly, only parenchyma cells that surround vascular bundles undergo lignification. This implies that vascular bundles might play essential roles in flesh lignification in loquat fruits during postharvest storage. In expanding tomato fruits, the transcriptome landscape across specific tissues showed that the vascular bundles had significantly more transcripts associated with transport and in response to stress compared with other transcripts and contained a great number of tissue-specific transcription factors, such as AP2/ERF, MYB, NAC, MADS, and WRKY transcript factors, while none were detected in parenchyma cells^49^. Some members of the MYB, NAC, and AP2/ERF families have been reported to be involved in lignification in postharvest loquat fruits. In sugarcane stem internodes, labeling of ShSUT1, a sucrose transporter, was detected in parenchyma cells surrounding vascular bundles; the surrounding cells then became lignified and suberized as stem development progressed, forming a barrier to apoplastic solute movement^50^. The results of these studies imply the potential function of vascular bundles to make lignify surrounding parenchyma cells. However, the specialized functions of vascular bundles in terms of lignification in loquat fruits during postharvest storage need to be further clarified.

It is of high interest to further investigate the exact molecular mechanism governing the spatiotemporal diversity of lignification in loquat fruits. In previous studies, we revealed that the activity and transcript levels of lignin biosynthesis-related enzymes, including L-phenylalanine ammonia lyase (PAL), cinnamate 4-hydroxylase (C4H), 4-coumarate: coenzyme A ligase (4CL), and cinnamyl alcohol dehydrogenase (CAD), were positively correlated with loquat fruit lignification during postharvest storage^15,44,51^. EjCAD1, EjCcoAOMT, and Ej4CL1 have been identified as key candidates involved in the regulation of chilling injury-related lignification^41,51,52^. Given that loquat lignification is highly tissue/cell-specific, the preferential expression of some transcription factors involved in lignin biosynthesis might explain the distinct patterns of lignin deposition in loquat fruits during development and postharvest storage. A novel MYB transcription factor, EjODO1, was recently reported to be involved in regulating lignin biosynthesis of young loquat fruits during development. However, in mature ripe fruits and during their subsequent storage, EjODO1 expression was not detected^53^. Several other transcription factors, such as EjMYB1, EjMYB2, EjMYB8, EjNAC1, and EjNAC3, were found to have transcriptional effects on the promoters of lignin biosynthesis-related genes involved in flesh lignification of loquat fruits during postharvest storage^16,22,54^. Therefore, the distinct temporal preferential expression of specific transcription factors might play a role in tissue/cell-specific deposition in loquat fruits during development and postharvest storage. However, the previous molecular analyses concerning loquat fruit lignification used pulverization and homogenization of fruit tissues for use as analytes. Consequently, no detailed spatial information about lignin biosynthesis gene expression was provided. In future works, the exact molecular mechanism governing the precise spatiotemporal deposition of lignin in loquat fruit flesh will be investigated.

In this study, patterned deposition of lignin was investigated not only in loquat flesh but also in Nicotiana and Arabidopsis stems. One reason for the use of Nicotiana and Arabidopsis was to demonstrate the reliability of the synthesized CA-Alk biocompatibility with lignification *in planta*. Another reason was to obtain a good understanding of lignification in loquat fruits. We sought to determine whether the lignification patterns in loquat flesh cells and Nicotiana and Arabidopsis stem cells display differences or share commonalities, especially the latter two are important model plant species. The results show that, in addition to differences, there were also some commonalities. For example, the imaging results of LCs in the S1 stage of loquat fruits show that both the inner wall layer and the outer periphery deposited newly formed lignin during incubation (Fig. 2). In Nicotiana, the tracheids showed a similar lignification pattern (Supplementary Fig. S3). This indicates that the double-sided accumulation of lignin in the highly lignified cells is not a phenomenon specific to loquat.

Cell wall lignification is a complex process in higher plants. It not only has essential roles in plant development but also is involved in responses to various biotic or abiotic stresses and thus performs important functions in the adaptation of plants to the environment. Although links between lignification and plant development or environmental stresses have been widely demonstrated, most studies have reported direct lignin content variation and, to a lesser extent, lignin composition and regulatory mechanisms. Very few works have involved in situ studies of the impact of lignification on cell wall structure formation and dynamics during specific plant development stages or in response to specific stresses. In Arabidopsis, Lee et al. revealed a novel honeycomb lignin structure that is closely associated with the precise separation of floral organs^3^. In this study, distinct lignification patterns were observed among different cell types in Arabidopsis and Nicotiana stems and among different flesh cells of loquat fruits at different stages, forming specific patterned cell/cell wall structures. Especially in the flesh of postharvest loquat fruits, new lignin was specifically deposited in the corners and middle lamellae of the parenchyma cells around the vascular bundles, resulting in the development of reticular structures. The formation of these reticular structures might provide support for the flesh and thus increase fruit firmness. Although postharvest flesh lignification induced by senescence or chilling stress is undesired by consumers, with respect to loquat fruits specifically, patterned lignification might be a positive response to senescence or chilling stress to survive nutrient deficiency or low-temperature environments.

Given the complexity and diversity of lignification *in planta*, it is important to precisely determine the location of the spatiotemporal deposition of lignin during plant development or in response to environmental stress, yet the use of pulverized and homogenized plant tissues would result in an average variation or could even mask the variation in lignification. The display of the precise patterned lignification dynamics *in planta* in response to specific stress or during a specific developmental period can help us explicitly target cells/tissues for further investigation, thus allowing us to explore the single-cell biological mechanism underlying lignification *in planta*.

**Fig. 5:**
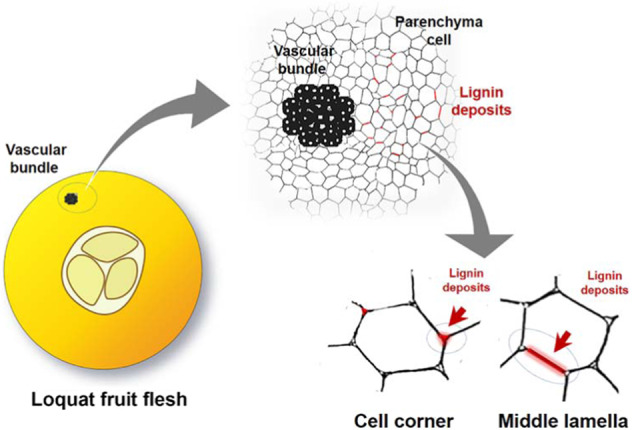
Tentative scheme for lignin deposition in loquat fruit flesh during storage. The newly formed lignin is specifically deposited in the cell corners and middle lamellae of parenchyma cells around the vascular bundles during postharvest storage

In fleshy fruits, the edible pulp is mainly formed by parenchyma cells with thin primary cell walls. The mechanical traits of parenchyma cell walls, together with water loss and cell turgor, are the main determinants of fleshy fruit firmness. In contrast to fruit softening, lignification led to an increase in lignin content and flesh firmness of postharvest loquat fruits. Here, we proposed a tentative scheme for lignin deposition in the flesh of loquat fruits during storage (Fig. 5). The newly formed lignin is specifically deposited in the cell corners and middle lamellae of parenchyma cells around the vascular bundles during postharvest storage. The patterned deposition of the newly formed lignin in the cell corners and middle lamellae in association with the parenchyma cells composes a reticular structure centered on the VB. This reticular structure might further provide mechanical support for the fleshy tissue and result in the hardening of the fruit.

## Materials and methods

### Plant materials and chemicals

Fruits of Luoyangqing loquat at the S1 stage (fruitlets), at 75 days after full bloom (DAFB), at the S2 stage (turning), at 100 DAFB, and at the S3 stage (maturity), and at 115 DAFB (Supplementary Fig. S4), were collected from an orchard at Luqiao, Zhejiang Province, in 2018 and 2019. *Arabidopsis thaliana* and *Nicotiana benthamiana* were grown in growth chambers, and stems of 6-week-old seedlings were obtained for experiments.

Coniferyl alcohol (CA) was purchased from Sigma (Shanghai, China). 3-O-propargylcaffeyl alcohol (CA-Alk) was synthesized according to previously reported procedures^37^. CA-Alk contains a bioorthogonal alkynyl functional group at the 3-O-position, which does not alter the fidelity of lignification^37^. Alexa 594-azide and Murashige and Skoog salts were purchased from Thermo Fisher Scientific (Life Technologies, Shanghai, China) and PhytoTechnology Laboratories (Lenexa, KS, USA), respectively. Other commercial chemicals, including solvents, were obtained from Sigma (Shanghai, China).

### NMR and Raman spectroscopy of CA-Alk

To confirm the structure of CA-Alk, NMR and Raman spectroscopy were performed. NMR spectra of the synthesized CA-Alk were obtained using a Bruker AVANCE III 400 (Bruker Daltonic, Inc., Bremen, Germany) instrument. CA-Alk was dissolved in dimethyl sulfoxide (DMSO) during NMR spectrum collection. Raman spectra of CA and CA-Alk were collected using a Renishaw inVia Reflex Raman Microscopy instrument equipped with a 532-nm diode laser (Renishaw Plc., Wotton-Under-Edge, UK). Solid powders of CA and CA-Alk were placed on a slide covered with silver paper. The spectra were collected under a ×50 objective over the spectral range from 600 to 3000 cm^−1^, with an exposure time and laser power of 0.5 s and 25 mW, respectively. The Raman spectra of CA and CA-Alk were plotted using Origin 8.1 Pro software (OriginLab, Northampton, MA, USA).

### Incubation together with CA-Alk

To determine the biological compatibility of the synthesized CA-Alk, we first applied it for tracing the deposition of the newly formed lignin in the model plant species *Arabidopsis thaliana* ecotype Columbia (Col-0) and *Nicotiana benthamiana*. Middle portions of stems of 6-week-old Arabidopsis and Nicotiana plants were frozen in optimum cutting temperature (OCT) freeze medium and cryosectioned into 40-μm-thick sections using a Leica CM1950 cryostat. After washing with water to remove the freezing medium, the cryosections were then incubated together with 1 mL of liquid-sterile 1/2-strength MS media consisting of either 20 μM CA-Alk or 20 μM CA at 25 °C for 24 h, with gentle rocking. After incubation, the sections were washed four times with 1 mL of 1/2-strength MS media to remove the nonincorporated monomers and then used for subsequent labeling.

We then incubated the cubes of loquat fruit flesh together with CA-Alk. Small cubes of flesh (sizes of ~2× 2 × 2 mm, 4 × 4 × 4 mm, and 6 × 6 × 6 mm) were obtained at the equatorial region of S1-, S2-, and S3-stage fruits, respectively. The cubes of the flesh of the S1 and S2 fruits were incubated together with 2 mL of liquid-sterile 1/2-strength MS media consisting of either 100 μM CA-Alk or 100 μM CA at 25 °C for 24 h. For the S3 fruit, the cubes of flesh were incubated together with 2 mL of 1/2-strength MS media consisting of 100 μM CA-Alk at 0, 5, and 20 °C for 2 days to simulate the postharvest storage conditions. After incubation, the cubes were washed four times with 2 mL of 1/2-strength MS media to remove the nonincorporated monomers.

### Bioorthogonal click labeling

After incubation and washing, the Arabidopsis and Nicotiana stem sections were directly placed in 1 mL of click labeling solution consisting of 1 mM ascorbic acid, 1 mM CuSO_4_, and 0.5 μM Alexa 594-azide in liquid 1/2 MS media. Click labeling was performed at 25 °C in the dark for 1 h. Sections were then washed with 1 mL of 1/2-strength MS media (2×, 10 min), 1 mL of 70% MeOH (1×, 60 min), and 1 mL of 1/2-strength MS media (4×, 10 min) successively to remove the unbound fluorophores.

For bioorthogonal click labeling of the newly incorporated CA-Alk in the lignin in loquat fruit flesh, the incubated cubes were sectioned by hand to a thickness of approximately 500 μm, which included several layers of cells. The sections were washed with liquid 1/2-strength MS media (4×) to remove the nonincorporated monomers. The sections were then placed in 2 mL of click labeling solution consisting of 2 mM ascorbic acid, 1 mM CuSO_4_, and 1 μM Alexa 594-azide in liquid 1/2-strength MS media for labeling at 25 °C in the dark for 1.5 h. After click labeling, the sections were washed with 2 mL of 1/2-strength MS media (2×, 10 min), 2 mL of 70% MeOH (1×, 60 min), and 2 mL of 1/2-strength MS media (4×, 10 min) successively to remove the unbound fluorophores.

### Confocal microscopy

After labeling and washing, the sections were carefully mounted on glass slides in water, covered by a coverslip, and sealed with nail polish. Confocal microscopy visualization was performed with a Zeiss LSM 780 microscope (Carl Zeiss, Jena, Germany) using ×20 (numerical aperture [NA] 0.8) and ×63 (NA 1.4, oil immersion) objectives with the following excitation laser and emission filter: a 561-nm excitation laser with a 617/73 emission filter for azide-fluor 594 and a 405-nm excitation laser with 450/50 for lignin autofluorescence.

### Lignin staining

The staining of lignin at both the flesh tissue and microscopic levels in loquat fruits was performed as described previously^40^. The lignin in the flesh was stained with phloroglucinol–HCl (Wiesner reagent) and observed using a binocular stereomicroscope (Carl Zeiss, Oberkochen, Germany). The fruits were directly cut at the equatorial plane into halves with a knife by hand. One milliliter of 1% phloroglucinol ethanol solution was dripped evenly onto the flesh plane, followed by the addition of drops of concentrated HCl 3 min later to cause the Wiesner reaction. The images of the lignin-stained flesh were captured within 5 min using a binocular stereomicroscope.

For microscopic staining of lignin, the loquat fruit flesh was first cryosectioned at a thickness of approximately 100 μm. The sections were subsequently stained with phloroglucinol–HCl, and the staining procedures were the same as those used for flesh staining. The images of the lignin-stained sections were captured within 5 min using a light microscope (Olympus, Tokyo, Japan).

## Supplementary information

Revised Supporting Information
