# Supplementary material for: Biosynthetic labeling with 3-O-propargylcaffeyl alcohol reveals in vivo cell-specific patterned lignification in loquat fruits during development and postharvest storage
Source: Hortic Res. 2021 Mar 10;8:61. doi: 10.1038/s41438-021-00497-z (PMC7943773; doi:10.1038/s41438-021-00497-z)
Supplement: Supplementary file 1 — Revised Supporting Information [file 41438_2021_497_MOESM1_ESM.docx]

**Supporting Information**

**Fig. S1** Chemical structure of the monolignol and Alk-tagged monolignol. (a) coniferyl alcohol (CA). (b) Alk-tagged coniferyl alcohol (CA-Alk). (c) Raman spectra of CA and CA-Alk. An additional peak attributed to Alk group was detected at 2128 cm^-1^. (d), NMR spectra of CA-Alk.

**Fig. S2** Incorporation of CA-Alk into stem sections of 6-week-old Nicotiana. The newly deposited lignin (561 nm, click labeling) were successfully highlighted and distinguished from the preexisted lignin (405 nm, lignin autofluorescence) in the stem sections after feeding with CA-Alk. Three biological replicates were set. Each biological replicate has at least three replicate sections imaged under each treated condition. Scale bar, 50 μm.

**Fig. S3** Patterned deposition of lignin in Nicotiana stem sections. Xylem, tracheid and cambium cells showed the 561 nm fluorescence signal indicating newly deposited lignin in these cells during incubation. Enlarged views show distinct lignin deposition modes for xylem and tracheid, showing the xylem inner wall layers were active in lignin deposition whereas the tracheids deposited lignin at both the inner wall layer and the outer periphery regions (thick white arrow on the left picture). Fluorescence intensity profiling of the 561 nm across the neighboring cell wall region in the xylem and tracheid (thin arrowhead) shows additional two neighboring but separated peaks (thick white arrow on the right picture) in the tracheid fluorescence curve compared with the xylem, indicating it’s the primary wall tightly adjacent outer periphery region, not the middle lamella, of the tracheid were active in lignification. Three biological replicates were set. Each biological replicate has at least three replicate sections imaged under each treated condition. Scale bar, 50 μm

**Fig. S4** Image of loquat fruit of different developmental stages and the flesh microstructures. S1, fruitlet; S2, turning stage fruit; S3, mature fruit. VB indicates the vascular bundle, PC indicates the parenchyma cell, LC indicates the lignified cell, taking S1 stage fruit as an example. Scale bar, 75 μm


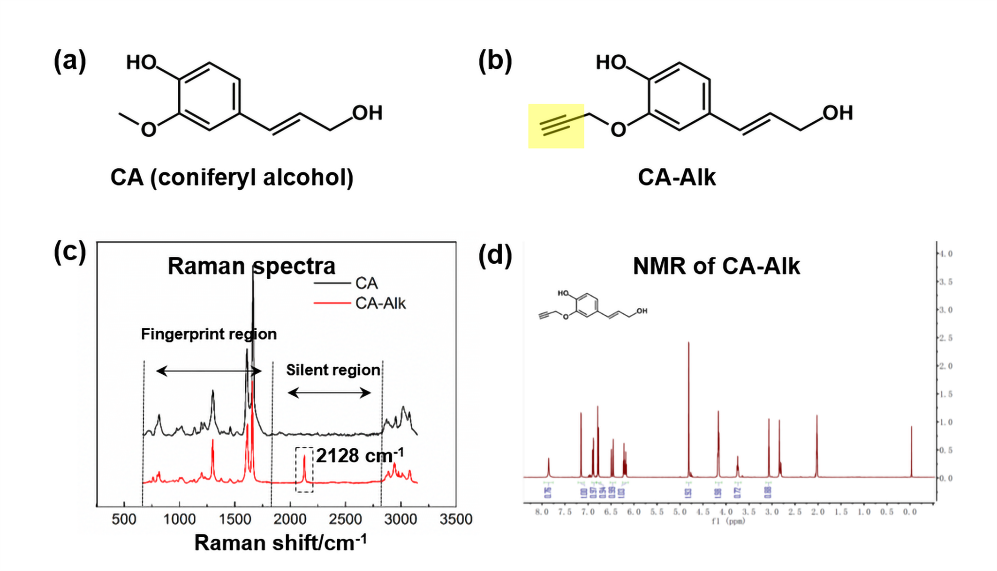


Fig. S1 Chemical structure of the monolignol and Alk-tagged monolignol. (a) coniferyl alcohol (CA). (b) Alk-tagged coniferyl alcohol (CA-Alk). (c) Raman spectra of CA and CA-Alk. An additional peak attributed to Alk group was detected at 2128 cm^-1^. (d), NMR spectra of CA-Alk.


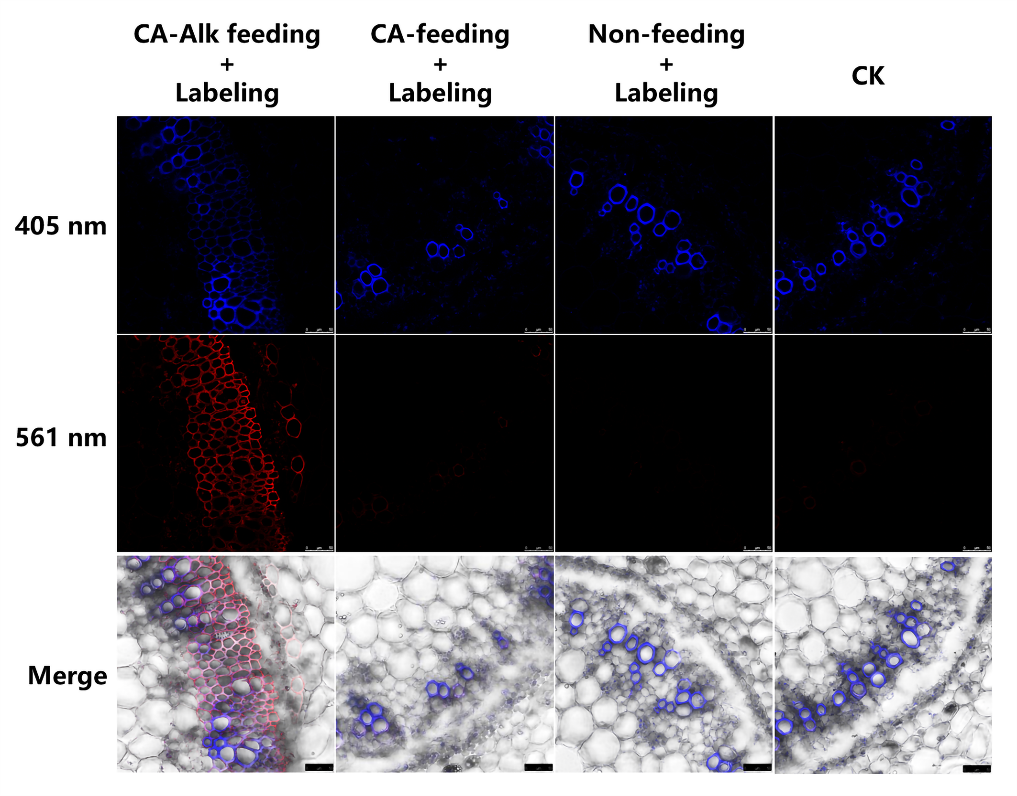


Fig. S2 Incorporation of CA-Alk into stem sections of 6-week-old Nicotiana. The newly deposited lignin (561 nm, click labeling) were successfully highlighted and distinguished from the preexisted lignin (405 nm, lignin autofluorescence) in the stem sections after feeding with CA-Alk. Three biological replicates were set. Each biological replicate has at least three replicate sections imaged under each treated condition. Scale bar: 50 μm.


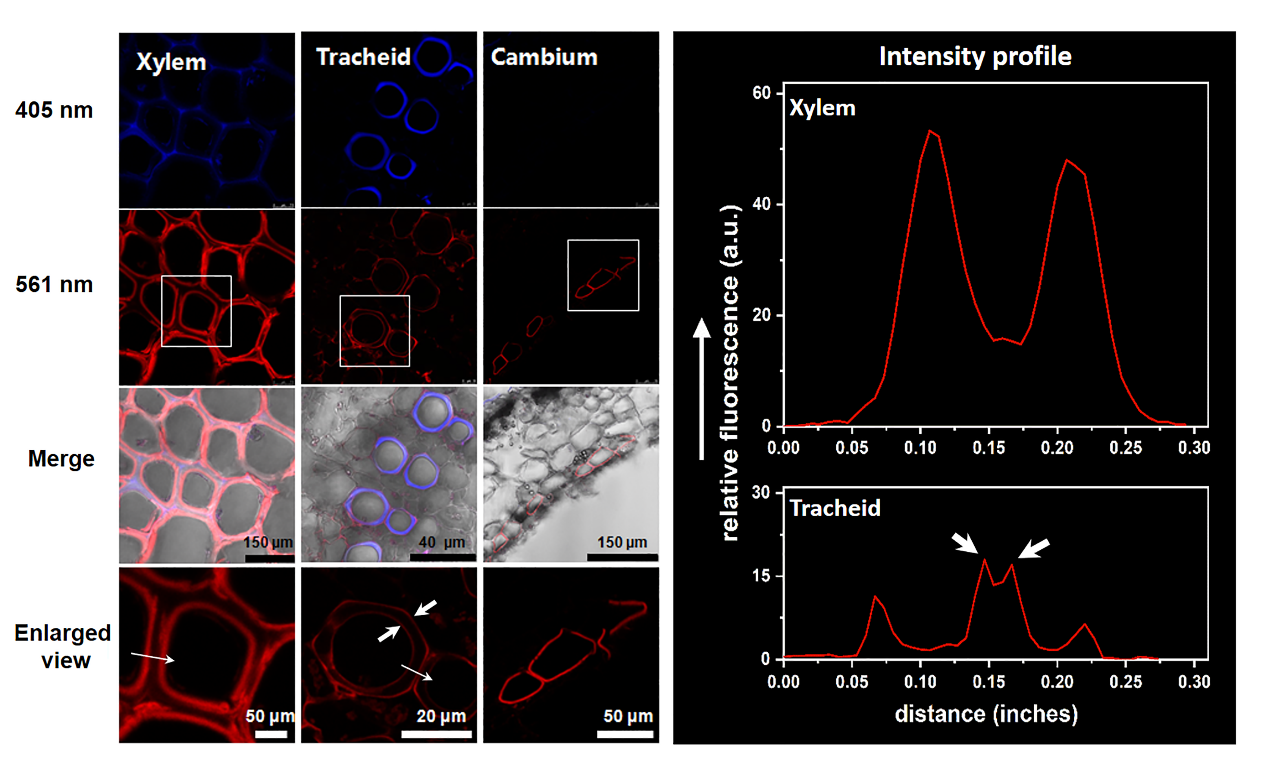


Fig. S3 Patterned deposition of lignin in Nicotiana stem sections. Xylem, tracheid and cambium cells showed the 561 nm fluorescence signal indicating newly deposited lignin in these cells during incubation. Enlarged views show distinct lignin deposition modes for xylem and tracheid, showing the xylem inner wall layers were active in lignin deposition whereas the tracheids deposited lignin at both the inner wall layer and the outer periphery regions (thick white arrow on the left picture). Fluorescence intensity profiling of the 561 nm across the neighboring cell wall region in the xylem and tracheid (thin arrowhead) shows additional two neighboring but separated peaks (thick white arrow on the right picture) in the tracheid fluorescence curve compared with the xylem, indicating it’s the primary wall tightly adjacent outer periphery region, not the middle lamella, of the tracheid were active in lignification. Three biological replicates were set. Each biological replicate has at least three replicate sections imaged under each treated condition. Scale bar, 50 μm


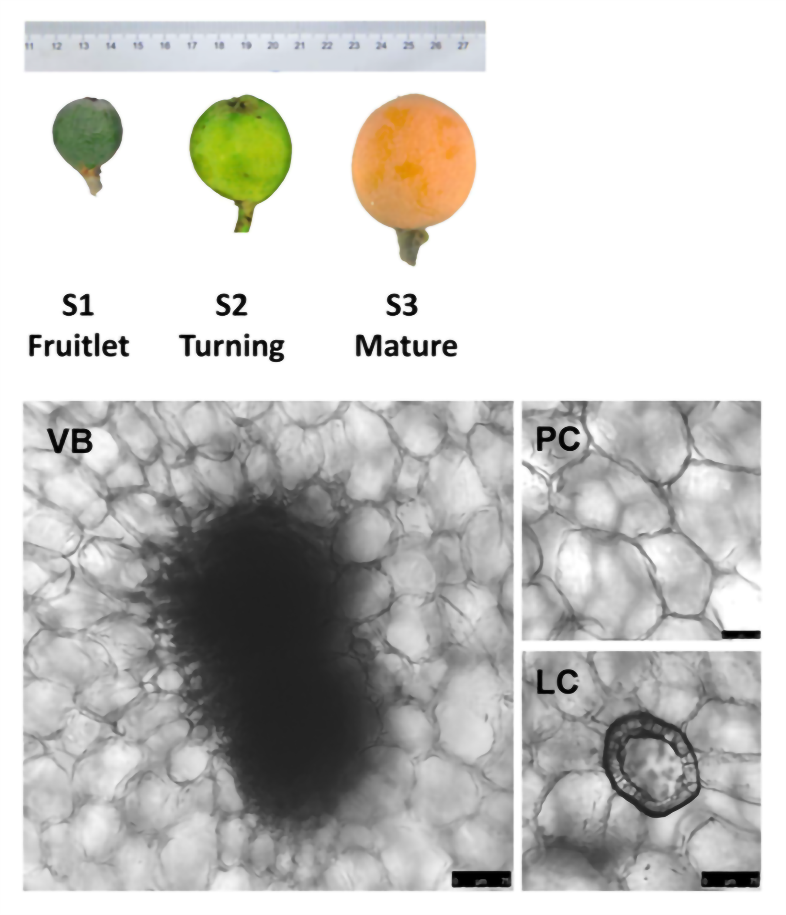


Fig. S4 Image of loquat fruit of different developmental stages and the flesh microstructures. S1, fruitlet; S2, turning stage fruit; S3, mature fruit. VB indicates the vascular bundle, PC indicates the parenchyma cell, LC indicates the lignified cell, taking S1 stage fruit as an example. Scale bar, 75 μm
